# Supplementary material for: A Comprehensive Assessment of Ultraviolet-Radiation-Induced Mutations in Flammulina filiformis Using Whole-Genome Resequencing
Source: J Fungi (Basel). 2024 Mar 20;10(3):228. doi: 10.3390/jof10030228 (PMC10971301; doi:10.3390/jof10030228)
Supplement: Supplementary file 1 [file jof-10-00228-s001.zip › Supplementary Material S8/KEGG annotation/out/64381550635650.os/KO/out_map/map05016.html]

KEGG PATHWAY: Huntington's disease - Reference pathway


|  |  |
| --- | --- |
| **Huntington's disease - Reference pathway** |  |

[
Pathway menu
| Organism menu
| Pathway entry
| Show description
| User data mapping
]

Huntington disease (HD) is an autosomal-dominant neurodegenerative disorder that primarily affects medium spiny striatal neurons (MSN). The symptoms are choreiform, involuntary movements, personality changes and dementia. HD is caused by a CAG repeat expansion in the IT15gene, which results in a long stretch of polyglutamine close to the amino-terminus of the HD protein huntingtin (Htt). Mutant Htt (mHtt) has effects both in the cytoplasm and in the nucleus. In the cytoplasm, full-length mHtt can interfere with BDNF vesicular transport on microtubules. This mutant protein also may lead to abnormal endocytosis and secretion in neurons, because normal Htt form a complex with the proteins Hip1, clathrin and AP2 that are involved in endocytosis. In addition, mHtt affects Ca2+ signaling by sensitizing InsP3R1 to activation by InsP3, stimulating NMDAR activity, and destabilizing mitochondrial Ca2+ handling. The mHtt translocates to the nucleus, where it forms intranuclear inclusions. Nuclear toxicity is believed to be caused by interference with gene transcription, leading to loss of transcription of neuroprotective molecules such as BDNF. While mHtt binds to p53 and upregulates levels of nuclear p53 as well as p53 transcriptional activity. Augmented p53 mediates mitochondrial dysfunction.

|  |  |
| --- | --- |
| Reference pathway | 100% |
